# Ectopic Cecal Varices as a Cause of Lower Gastrointestinal Bleeding

**DOI:** 10.1155/2023/7005565

**Published:** 2023-06-15

**Authors:** Abdulrahman Qatomah, Sulaiman Almushir, Faisal Aljohani

**Affiliations:** ^1^Division of Gastroenterology and Hepatology, McGill University Health Centre, Montreal, QC, Canada; ^2^Division of Gastroenterology and Hepatology, King Faisal Specialist Hospital and Research Center, Jeddah, Saudi Arabia; ^3^Department of Internal Medicine, Tabuk University, Tabuk, Saudi Arabia

## Abstract

Ectopic varices account for 1%–5% of all variceal bleeding episodes in patients with portal hypertension. They can be found at any part of gastrointestinal tract including the small intestines, colon, or rectum. We report a case of a 59-year-old man who presented with bleeding per rectum 2 days after a routine colonoscopy, in which 2 lesions were biopsied. Gastroscopy was negative for bleeding, and he was not stable enough to undergo colonoscopy. CT angiography showed a large portosystemic shunt with multiple collaterals in the right lower quadrant. These findings were clues for a diagnosis of ectopic cecal varices.

## 1. Introduction

Gastrointestinal bleeding from gastric or esophageal varices is common in patients with portal hypertension; however, ectopic varices constitute about 1%–5% of all variceal bleeding episodes in patients with portal hypertension. [1]. Ectopic varices can be classified as luminal or extra-luminal. Luminal varices can be found at any part of the gastrointestinal tract including the small intestines, colon, rectum, and surgical ostomy sites, while extra-luminal varices can be found in the peritoneal space, gallbladder, or ovaries [2]. Fewer than 100 cases of colonic varices have been reported in the literature which reflect how uncommon they are. Ectopic varices represent a clinical challenge given how rare they are, possibly been underdiagnosed, underreported, and the fact that they may bear a resemblance in the endoscopic appearance to other common endoscopic pathologies. Currently, the management of ectopic varices is based largely on case reports and expert opinion with no clear guidelines on the management.

Cecal varices are considered a rarer entity and most commonly present as a massive lower gastrointestinal hemorrhage. However, in extremely rare conditions, cecal varices may be found on routine colonoscopies without symptoms and may have the appearance of submucosal lesions without the classical appearance of varices that are usually seen on upper endoscopy. Over-insufflation can cause the collapse of the varices which lead to inaccurate diagnosis of the varices. There are only 2 cases reported in the literature of asymptomatic cecal varices [1, 3–5]. Cecal varices develop because of either cirrhotic or noncirrhotic portal hypertension, mesenteric vein thrombosis, biliary atresia, or biliary sclerosis [1, 3, 5].

## 2. Case Report

**Figure 1:**
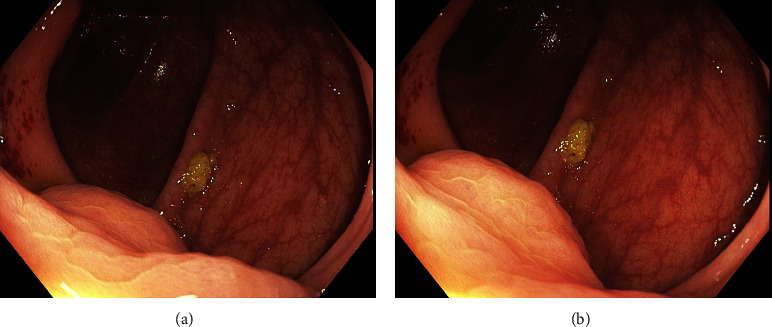
(a, b) Endoscopic image with semi-circumferential lesion just above the ileocecal valve without the typical appearance of varices.

A 59-year-old male who is known for compensated alcoholic liver cirrhosis presented to the emergency department with one-day history of bright red blood per rectum 2 days after routine colonoscopy for a positive occult stool test. He did not have any upper gastrointestinal bleeding symptoms, and his colonoscopy prior to this presentation only showed mild pan-diverticulosis and a single serrated polyp measuring 6 mm removed by cold snare in the transverse colon. Also, a semi-circumferential lesion just above ileocecal valve was noted which was biopsied (Figures 1(a) and 1(b)). The patient has a remote history of prior esophageal variceal bleeding treated with nonselective beta blockers and repeated banding. Additionally, he has a remote history of a lower gastrointestinal bleeding which was initially managed with endoscopic placement of hemostatic clips followed by angio-embolization of ileocolic artery, a branch of superior mesenteric artery, and this bleeding is thought to be arterial in origin. His most recent gastroscopy 2 years prior to this presentation showed small esophageal varices without high-risk stigmata. The patient has been completely abstinent from alcohol.

**Figure 2:**
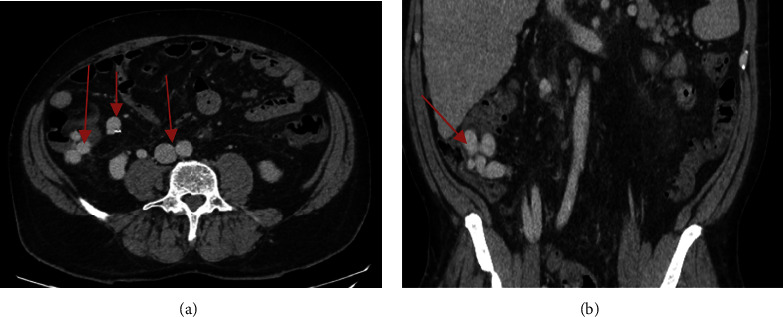
Abdomen CT ((a) axial and (b) coronal) showing portosystemic shunt with multiple collaterals in the right lower quadrant, in between the superior mesenteric vein and inferior vena cava (red arrows).

**Figure 3:**
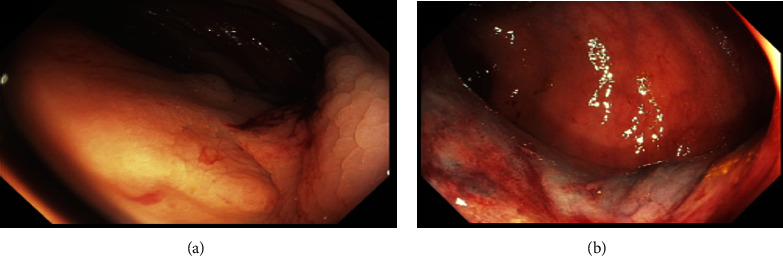
(a, b) Endoscopic image showing the cecum after sclerotherapy (thrombin) with no evidence of active bleeding.

Initially, the patient was stable. However, he had significant drop of hemoglobin by 20 gram per liter from baseline. He underwent gastroscopy, which showed small esophageal varices without high-risk stigmata. No gastric varices or evidence of bleeding was found. He was prepped for a colonoscopy the next morning. He continued to bleed overnight and became hemodynamically unstable requiring multiple blood transfusions. Decision was made to proceed with computed tomography (CT) angiography which showed no evidence of intra-abdominal hemorrhage or active contrast extravasation. However, there are large portosystemic shunts with multiple collaterals in the right lower quadrant, in between the superior mesenteric vein and inferior vena cava, with embolization coils in the right lower quadrant and no evidence of thrombosis on mesenteric or portal veins (Figures 2(a) and 2(b)). The diagnosis of bleeding ectopic cecal varices was made. The bleeding was thought to be related to biopsy from lesion that had been seen on the colonoscopy, which was ectopic cecal varix. The patient was started on prophylactic antibiotics, octreotide, and underwent angio-embolization of the ectopic cecal varices by interventional radiology. However, he continued to bleed requiring multiple transfusions. Subsequently, he underwent transjugular intrahepatic portosystemic shunt (TIPS) after measuring the hepatic venous pressure gradient (HVPG), which was found to be elevated at 15 mmHg. His condition stabilized and prior to be discharged, he bled again. He underwent a colonoscopy, which showed bleeding just above the ileocecal valve at biopsy site, which was treated with sclerotherapy (thrombin), and hemostasis was achieved (Figures 3(a) and 3(b)). The patient was monitored for few days, and then he was discharged home.

## 3. Discussion

In our case, the initial colonoscopy showed a semi-circumferential lesion just above the ileocecal valve without the typical appearance of varices that are usually encountered on upper endoscopy which was a cecal varix (Figures 1(a) and 1(b)). It is possible that the prior angio-embolization of an arterial bleeding in the same area of variceal bleeding contributes in creating a high venous pressure that contributes in the development of cecal varix; however, it is very difficult to confirm such hypothesis.

Up to the date of writing this case report, there are no existent guidelines regarding the management of ectopic varices. The current management is largely based on case reports and expert opinion. Endoscopic management with sclerotherapy has been reported to be successful. Another option would be angio-embolization of ectopic varices. However, none of these interventions would lead to the decompression of portal pressure. In fact, angio-embolization can lead to an increase in portal pressure. TIPS and liver transplant are considered definitive therapy in ectopic cecal varices secondary to cirrhotic portal hypertension [1, 4–7].

In the few cases that report refractory bleeding ectopic cecal varices, total and partial colectomy had been reported [4–6]. The use of vasoactive substances like octreotide and *β*-blockers has been tried but not studied.

Finally, this case illustrates the importance of maintaining high degree of suspicion for ectopic varices among patients with known portal hypertension whenever performing endoscopy and encountering atypical lesions to avoid performing biopsy or any endoscopic intervention if there is any suspicion that the lesion may represent ectopic varices. As the consequences of performing endoscopic intervention can lead to unforgivable life-threating complications, reviewing prior cross-sectional imaging with contrast should be done prior to endoscopic intervention to assess for the presence of ectopic varices or in cases of encountering endoscopic lesions that may represent ectopic varices. If there is no prior cross-sectional imaging available, then the next step is to obtain imaging prior to intervention or perform endoscopic ultrasound if feasible.

## Data Availability

All data generated or analyzed during this study are included in this article. Further enquiries can be directed to the corresponding author.
